# Supplementary material for: Accuracy and Reproducibility of Contrast-Enhanced Mammography in the Assessment of Response to Neoadjuvant Chemotherapy in Breast Cancer Patients with Calcifications in the Tumor Bed
Source: Diagnostics (Basel). 2021 Mar 4;11(3):435. doi: 10.3390/diagnostics11030435 (PMC7999407; doi:10.3390/diagnostics11030435)
Supplement: Supplementary file 1 [file diagnostics-11-00435-s001.pdf]

Supplementary Materials

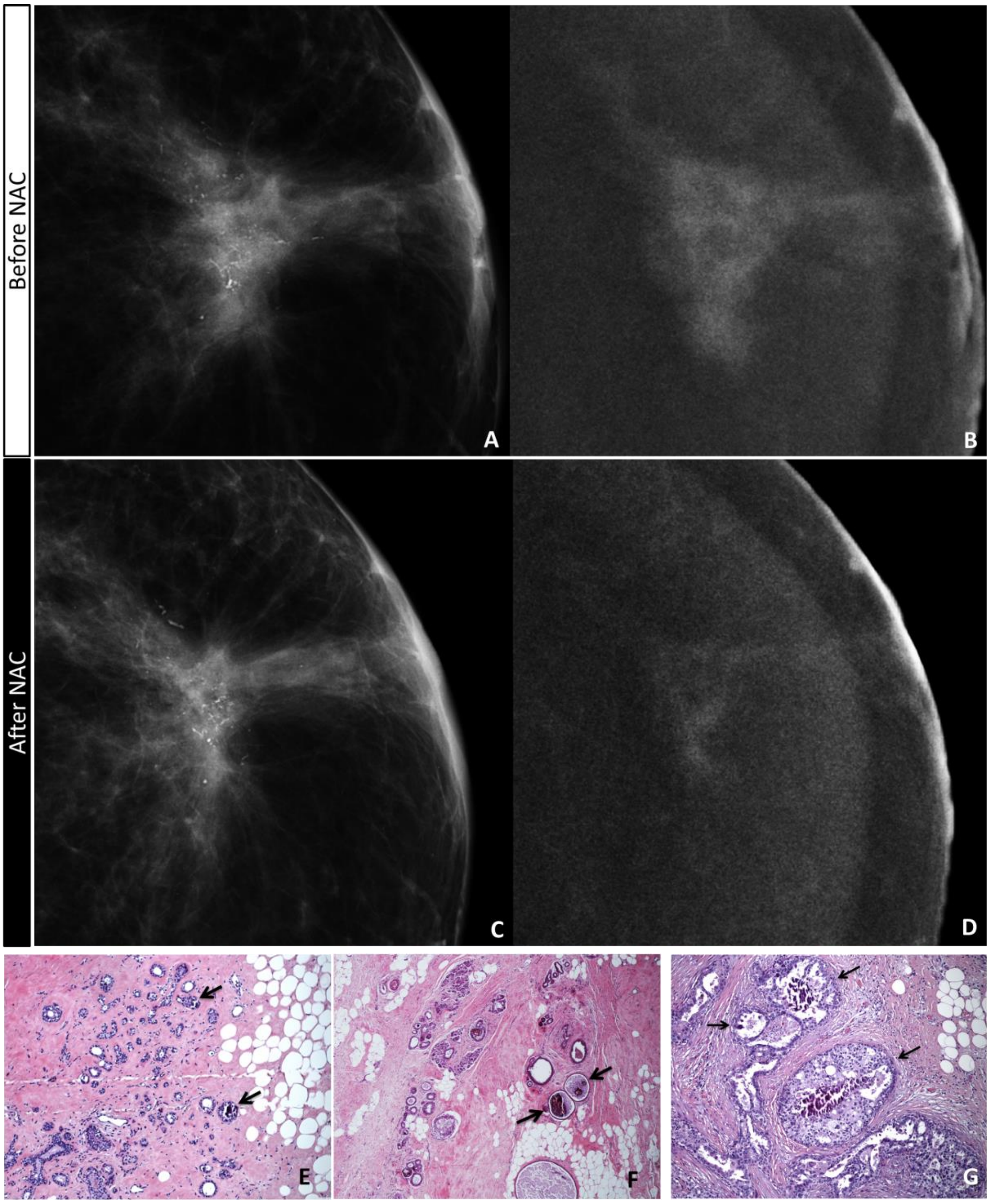

Figure S1. 36-yo woman with IDC G2, luminal B, presenting pre-NAC with almost 50 mm predominantly pleomorphic calcifications (**A**: CC low-energy image) and 40 mm mass enhancement (**B**: recombined image). After NAC, the calcifications were considered stable (**C**: CC low-energy image), with a concentric shrinkage of enhancement (**D**: CC recombined image). Analyzing the surgical specimen, calcifications were detected in benign ducts (**E**,**F**) and associated with ductal carcinoma in situ (**G**). E: amorphous calcifications (thick arrows) associated with adenosis (4×); F: amorphous calcifications (thick arrows) associated with ectatic ducts (4×). G: amorphous calcifications (thin arrows) present in the luminal of ductal carcinoma in situ, moderately differentiated, cribriform type (10×).

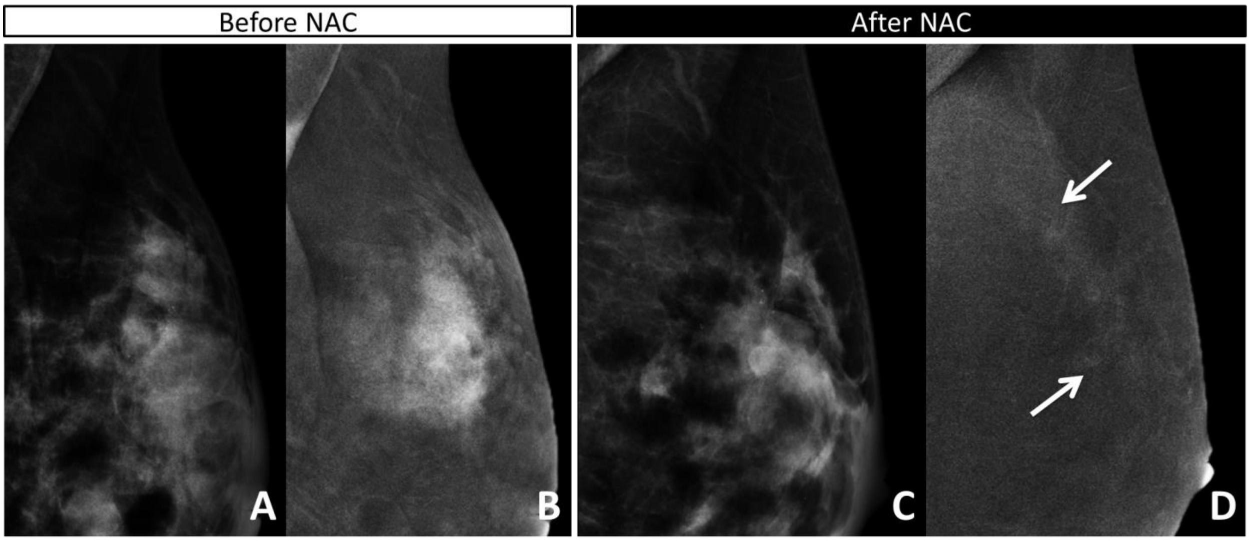

Figure S2. False positives for both the combined and the CE evaluation: 43-yo woman with IDC G2 + DCIS HER2+ with a glandular thickening in the outer quadrant of left breast and inner pleomorphic calcific component (**A,C**: MLO low-energy image). The thickening showed a strong non-mass enhancement before NAC (B: MLO recombined image). After NAC (**C**: MLO low-energy image; **D**: MLO recombined image), the calcifications were stable, still pleomorphic, thus considered pathological. Some very faint foci of enhancement were described in the tumor bed (**D**, arrows), also considered pathological. The analysis of the surgical specimen revealed a complete response ypT0.

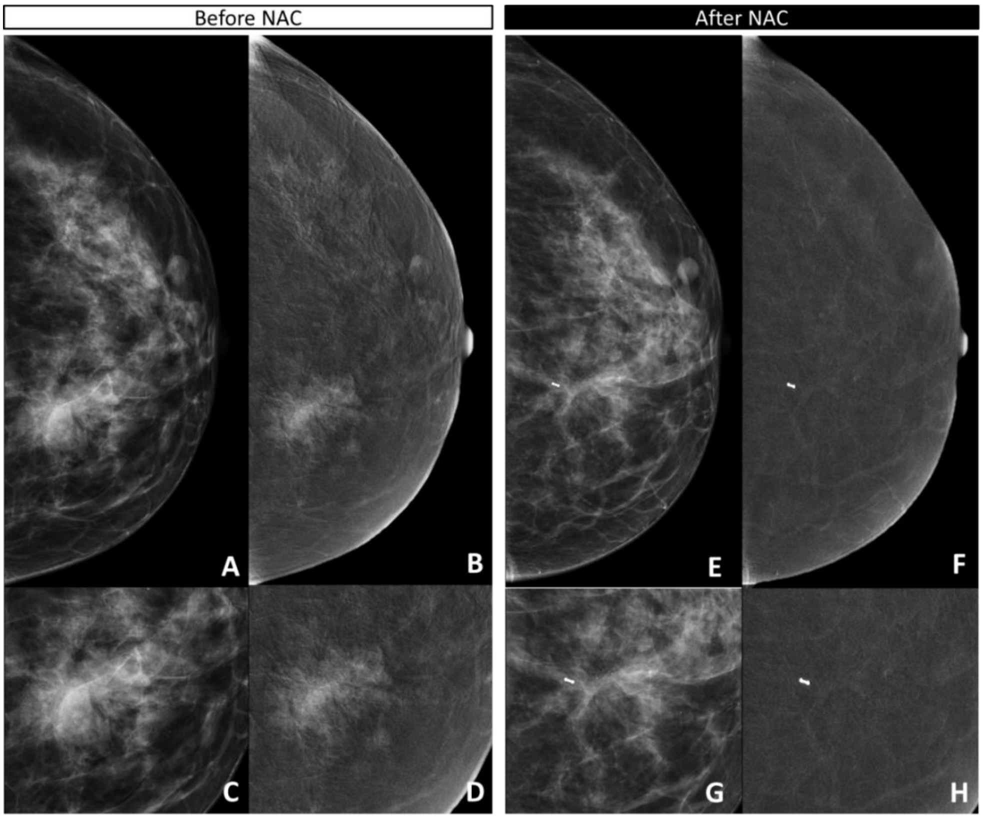

Figure S3. False negative for both the combined evaluation and the CE evaluation: 50-yo woman with IDC G2, luminal B HER2+, with irregular opacity with speculated margins and inner indeterminate calcifications in left breast. The in situ component was described only on the surgical specimen. The opacity showed a strong mass enhancement before NAC. After NAC, the indeterminate calcifications were stable and considered non-suspicious; no residual enhancement was visible surrounding the marker placed in the tumor bed of the opacity despite some still visible parenchymal distortion. The analysis of the surgical specimen revealed 15 mm of residual IDC and DCIS (ypT1c).
